# Apert Syndrome With FGFR2 758 C > G Mutation: A Chinese Case Report

**DOI:** 10.3389/fgene.2018.00181

**Published:** 2018-05-17

**Authors:** Yahong Li, Dingyuan Ma, Yun Sun, Lulu Meng, Yanyun Wang, Tao Jiang

**Affiliations:** Center of Prenatal Diagnosis, The Affiliated Obstetrics and Gynecology Hospital of Nanjing Medical University, Nanjing Maternity and Child Health Care Hospital, Nanjing, China

**Keywords:** Apert syndrome, craniosynostosis, FGFR2, genetic mutation, exons sequencing

## Abstract

**Background:** Apert syndrome is considered as one of the most common craniosynostosis syndromes with a prevalence of 1 in 65,000 individuals, and has a close relationship with point mutations in FGFR2 gene.

**Case report:** Here, we described a Apert syndrome case, who was referred to genetic consultation in our hospital with the symptom of craniosynostosis and syndactyly of the hands and feet. Craniosynostosis, midfacial retrusion, steep wide forehead, larger head circumference, marked depression of the nasal bridge, short and wide nose and proptosis could be found obviously, apart from these, ears were mildly low compared with normal children and there was no cleft lip and palate. Mutation was identified by sanger sequencing and a mutation in the exon 7 of FGFR2 gene was detected: p.Pro253Arg (P253R) 758 C > G, which was not found in his parents.

**Conclusion:** The baby had Apert syndrome caused by 758 C > G mutation in the exon 7 of FGFR2 gene, considering no this mutation in his parents, it was spontaneous.

## Background

Apert syndrome (AS) is considered to be one of the most common craniosynostosis syndromes (Fernandes et al., 2016). AS is closely related with the mutations in the gene of fibroblast growth factor receptor 2 (FGFR2), and the occurrence of the mutations increases with the age of the father (Yoon et al., 2009; Lumaka et al., 2014). The prevalence of AS is 1/ 65,000 in general population (Giancotti et al., 2014). AS follows dominant genetic patterns, and most patients are *de novo* cases caused by mutations of FGFR2 (Liu et al., 2013).

AS patients have unique clinical features such as craniosynostosis, midfacial malformations, syndactyly of the hands and feet (Pettitt et al., 2017). However, Coomaralingam et al (Coomaralingam and Roth, 2012) reported an AS confirmed by molecular genetic analysis, but the patient didn’t have craniosynostosis at birth. AS patients may have oral problems such as severe maxillary hypoplasia, cleft lip and cleft palate (Slaney et al., 1996; Kakutani et al., 2017). Other complications are commonly observed in AS patients such as conductive hearing loss, central nervous system anomalies, intellectual disability, autism and visual impairment (Morey-Canellas et al., 2003; Da et al., 2005; Rajenderkumar et al., 2005; Khong et al., 2007). Cohen et al. reported visceral abnormalities such as cardiac and gastrointestinal in AS (Cohen and Kreiborg, 1993).

FGFR2 gene mutations have significant association with AS. FGFR2 is the receptor of fibroblast growth factor (FGF) which is encoded by a gene at locus 10q26. FGFR2 is activated by binding to FGF and plays a role in cell proliferation, angiogenesis, bone differentiation and so on (Dalben et al., 2006; Lunn et al., 2007; Yeh et al., 2016). Mutation of the exon IIIa of FGFR2 can cause AS because this mutation leads to increased bone differentiation rate of MSCs (Mesenchymal Stem Cells) and the development of craniosynostosis. Common types of gene mutation are FGFR2 p.Ser252Trp (S252W) of 755C > G and p.Pro253Arg (P253R) of 758C > G. S252W mutation of FGFR2 is usually accompanied by severe skeletal malformations of craniofacial and higher incidence of cleft palate, but P253R mutation of FGFR2 is often accompanied by more prominent syndactyly of hands and feet (Slaney et al., 1996). In addition, abnormal visual acuity, nasolacrimal duct obstruction, astigmatism and strabismus are more obvious in patients with S252W mutation (Jadico et al., 2006). These two types of mutations account for 98% of AS, and there are other genotypes reported in AS, such as gene deletion, Alu element insertion, heterozygous mutation and sequence variation (Torres et al., 2015).

There are fewer reports on AS from China, so we reported a case of AS and analyzed the type of gene mutation in order to provide more characteristics and information of AS patients in China. It can assist in the diagnosis of AS for Chinese clinicians.

## Case Presentation

**FIGURE 1:**
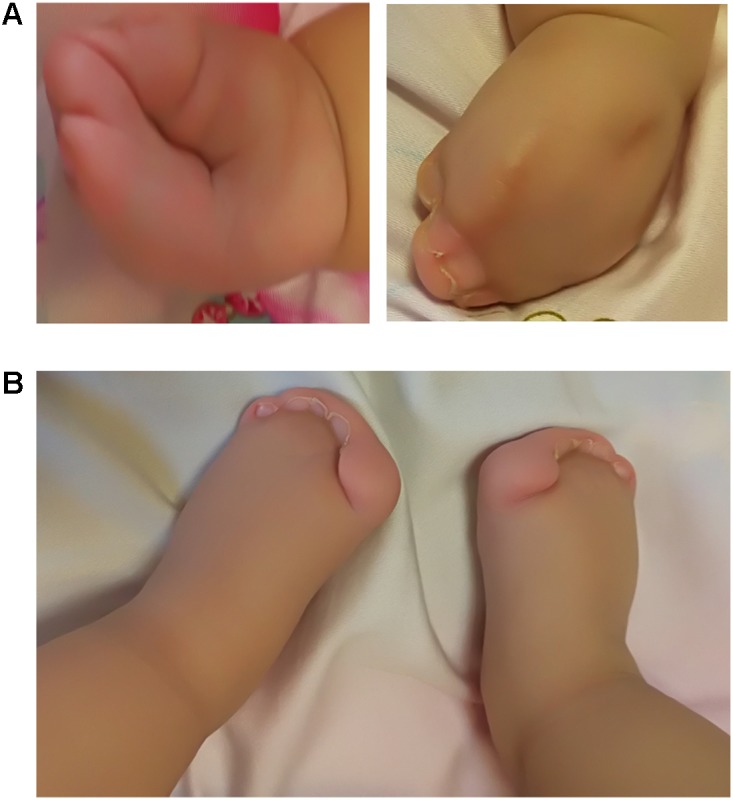
Syndactylies and contraction of the hands and feet. **(A)** Both sides of the hands; **(B)** Severe syndactyly of toes.

**FIGURE 2:**
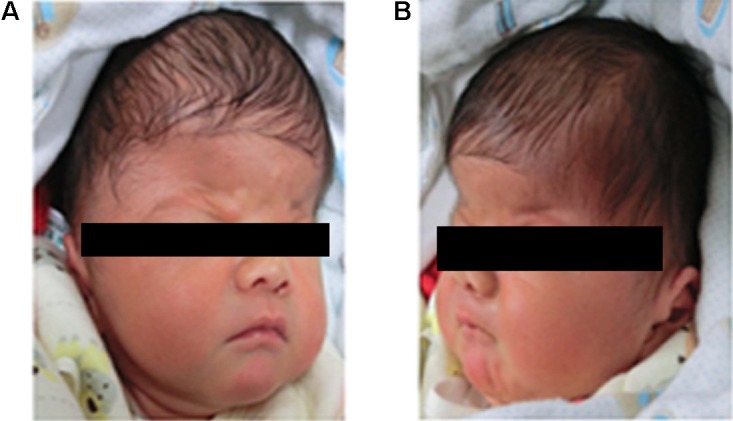
Head features of the Apert syndrome patient. **(A)** Craniosynostosis, midfacial retrusion, steep wide forehead, larger head circumference, marked depression of the nasal bridge, short and wide nose and proptosis could be found obviously; **(B)** Ears were mildly low compared with normal children.

**FIGURE 3:**
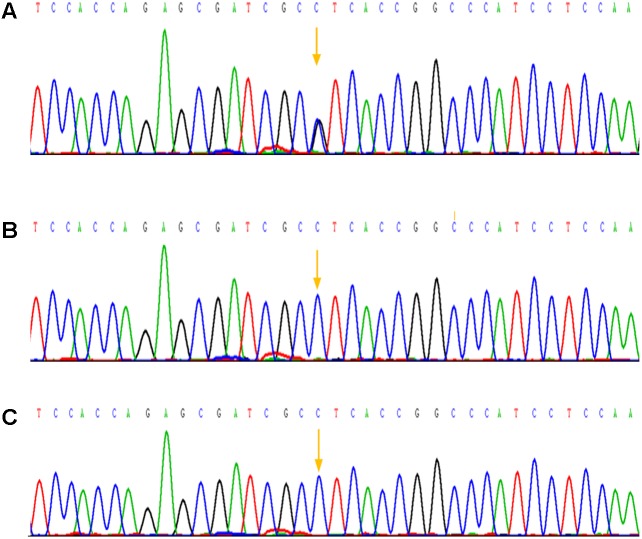
Mutation analysis of the Apert syndrome patient and his parents. **(A)** A mutation in the sequence of exon 7 was detected: p.Pro253Arg (P253R) 758 C > G; **(B)** The sequence of exon 7 of his mother and no mutation was detected; **(C)** The sequence of exon 7 of his father, as well there was no mutation was detected.

A 4-days-old male baby was referred to genetic consultation in our hospital with the symptom of craniosynostosis and syndactyly of the hands and feet. He was the first child of healthy non-consanguineous Chinese parents, and born at 38+2 weeks of gestation by cesarean section delivery after an uneventful pregnancy. At birth, his weight was 3500 g, length 52 cm and Apgar scores were 9 at 1 and 10 at 5 min. Specific symptoms of this AS were as follows: (1) syndactylies and contraction of the hands (**Figure 1A**) and feet (**Figure 1B**); (2) craniosynostosis, midfacial retrusion, steep wide forehead, larger head circumference, marked depression of the nasal bridge, short and wide nose and proptosis (**Figure 2A**); (3) the ears were mildly low compared with normal children, and there was no cleft lip and palate (**Figure 2B**). His mother and father were 25 and 30 years old, respectively, who came from Yancheng, a city of Jiangsu province, and both parents had no obvious body abnormalities, furthermore, the mother did not find any complications during pregnancy and delivery.

In addition to that, other physical examination of the patient was normal. Based on the above clinical symptoms, we highly suspected that he had AS. We obtained peripheral blood of the patient and his parents, and genomic DNA was extracted by using an Automated Nucleic Acid Extractor. Amplification of Exon 7 and Exon 9 by polymerase chain reaction (PCR), and PCR products were purified after agarose gel electrophoresis, then DNA sequence analysis was performed on the ABI3130 sequencer to determine FGFR2 gene mutations. As a result, the C.758C > G mutation in the coding region of exon 7 of the FGFR2 gene in the patient was confirmed (**Figure 3A**). A C-to-G transversion at position 758 resulting in a proline-to-arginine substitution at codon 253 in the FGFR2 protein (p.P253R). However, none of the mother and the father of this patient had mutations at this site (**Figures 3B,C**), indicating that the mutation of this patient was spontaneous and not from parents.

## Discussion

S252W and P253R are common gene mutation types of FGFR2, although S252W variant is more common (Azoury et al., 2017). This present case showed a fetus affected by P253R mutation, and it often associated with severe syndactyly of the feet and hands, which is confirmed in this case. The absence of cleft lip and palate defects also supported P253R mutation in this case, because cleft lip and palate defects are considered to be more frequent in patients with S252W mutation (Slaney et al., 1996).

An autosomal dominant mode of inheritance has been confirmed for AS, but, most cases are sporadic (Mantilla-Capacho et al., 2005). In this case, P253R mutation was a new mutation which was not present in the FGFR2 gene of his parents, thus we consider it as a spontaneous mutation, and it may play an important role in the course of the disease. Cohen et al. reported that the probability of AS mutation in a single gene in each generation was about 7.8/10^6^ (Cohen et al., 1992), and a complex interaction of environment, epigenetics, and genetics may be involved in AS (Azoury et al., 2017). Because the cases of AS are rare, so reports for Chinese people are even more precious. We searched reports on FGFR2 mutations in AS patients of Chinese, and the following types were presented: S252W and P253R, no other types have been found yet (Chang et al., 1998; Tsai et al., 1999; Dai et al., 2010).

Based on craniosynostosis phenomenon in AS patients, fetal magnetic resonance imaging (MRI) and ultrasound could identify AS case in the second-trimester and the third-trimester of pregnancies. Abnormal cranial shape, midfacial hypoplasia, and bilateral syndactyly of the hands and feet are diagnostic criteria for AS. Fetal prenatal ultrasound results for the head of AS may include the forehead uplift, short head, sharp skull, irregular head shape, abnormal biparietal diameter and head circumference, and even cloverleaf skull in severe case (Weber et al., 2010). Furthermore, ventriculomegaly, hydrocephalus, thalamic fusion, changes in corpus callosum and other central nervous system abnormalities or organ abnormalities are usually present in AS (Quintero-Rivera et al., 2006; Ludwig et al., 2012). However, because of the limitations in prenatal imaging, many patients can’t be detected by ultrasound (Rubio et al., 2016). In particular, it is difficult to capture broad thumbs in prenatal imaging, the syndactyly of the hands may be considered as clenched hands. Although there are craniosynostosis and midfacial malformations in AS, they may be absent or very subtle in the second-trimester of pregnancy and become obvious only in the third trimester. Thus not all cases can be detected based on craniosynostosis and midfacial malformations. Because of the lack of standard criteria for these defects, fetal maxillofacial and profile anomalies are still based on subjective judgment.

In this research, we diagnosed a case of AS from China through analyzing the mutation of FGFR2 gene. We hope it can provide more information about AS for clinicians to make diagnosis.

## Ethics Statement

The genetic study was approved by Medical Ethics Committee of Nanjing Maternity and Child Health Care Hospital. We obtained written informed consent for genomic analysis from the parents of the baby and the mother of the patient provided written informed consent for the publication of this case report.

## Author Contributions

YL and DM participated in experiments and wrote this article. YS, LM, and YW were responsible for sample collection and information collection. TJ guided the entire essay.

## Conflict of Interest Statement

The authors declare that the research was conducted in the absence of any commercial or financial relationships that could be construed as a potential conflict of interest.
